# Rape Straw Supported FeS Nanoparticles with Encapsulated Structure as Peroxymonosulfate and Hydrogen Peroxide Activators for Enhanced Oxytetracycline Degradation

**DOI:** 10.3390/molecules28062771

**Published:** 2023-03-19

**Authors:** Guiyin Wang, Yan Yang, Xiaoxun Xu, Shirong Zhang, Zhanbiao Yang, Zhang Cheng, Junren Xian, Ting Li, Yulin Pu, Wei Zhou, Gang Xiang, Zhien Pu

**Affiliations:** 1 College of Environmental Sciences, Sichuan Agricultural University, Chengdu 611130, China; 2 College of Resources, Sichuan Agricultural University, Chengdu 611130, China; 3 College of Agronomy, Sichuan Agricultural University, Chengdu 611130, China

**Keywords:** organic pollutants, advanced oxidation, fenton/fenton-like process, singlet oxygen, wastewater treatment

## Abstract

Iron-based catalysts with high load content of iron sulfide (FeS) were commonly peroxymonosulfate (PMS) and hydrogen peroxide (H_2_O_2_) activators to degrade organic pollutants but limited catalytic efficiency and increased risk of ferrous ion leaching restricted their use. Meanwhile, various biomass materials such as straw, peel, and branch have been extensively prepared into biochar for mechanical support for iron-based catalysts; however, the preparation process of biochar was energy-intensive. In this study, FeS nanoparticles modified rape straw composites (RS–FeS) encapsulated with ethylenediaminetetraacetic acid (RS–EDTA–FeS) were successfully presented by in-situ synthesis method for efficiently activating PMS and H_2_O_2_ to degrade oxytetracycline (OTC), which was economical and environmentally friendly. The results showed that the modified rape straw can remove OTC efficiently, and the addition of EDTA also significantly enhanced the stability and the reusability of the catalyst. In addition, EDTA also promoted the activation of H_2_O_2_ at neutral pH. The OTC degradation efficiency of the two catalysts by PMS was faster than that of H_2_O_2_, but H_2_O_2_ had a stronger ability to remove OTC than PMS. The highest OTC removal efficiency of RS–FeS and RS–EDTA–FeS were 87.51 and 81.15%. O_2_^•–^ and ^1^O_2_ were the major reactive oxidative species (ROS) in the PMS system. Furthermore, compared with RS–FeS, the addition of EDTA inhabited the generation of O_2_^•–^ in the PMS system. Instead, O_2_^•–^ and ^•^OH were the major ROS in the H_2_O_2_ system, but ^1^O_2_ was also identified in RS–FeS/H_2_O_2_ system. RS–EDTA–FeS showed a trend of rising first and then decreasing in recycle test. Instead, the removal rate of OTC by RS–FeS decreased significantly with the increase in reuse times. In the actual wastewater test, the TOC removal of two catalysts active by H_2_O_2_ was better than PMS, which was consistent with the test results of OTC, indicating that the two catalysts have application value in the removal of organic pollutants in actual wastewater. This study directly used plant materials as catalysts and omits the preparation process of biochar, greatly reduces the preparation cost and secondary pollution of catalysts, and provides theoretical support for the deepening of advanced oxidation technology.

## 1. Introduction

With the vigorous development of the pharmaceutical industry, antibiotic pollution has become a global environmental problem. Antibiotics, such as tetracyclines, sulfonamides, macrolides, fluoroquinolones and lincosamides, have been frequently identified in surface water due to their stability and poor biodegradability [1,2]. As a tetracycline antibiotic, oxytetracycline (OTC) has been widely used in breeding to treat diseases because of its low price and good antibacterial property [3,4]. OTC inhibits the growth of microorganisms and increases the abundance of antibiotic resistance bacteria, posing a growing threat to the global ecosystem [3]. Therefore, there is an imperious demand to develop more efficient, economical, and environmentally friendly methods to remove OTC in water.

Peroxymonosulfate (PMS) based and hydrogen peroxide (H_2_O_2_) based advanced oxidation processes are deemed as preeminent means to remove refractory contaminants benefiting from the generation of highly reactive radicals (sulfate radical (SO_4_^•−^), hydroxyl radical (^•^OH), superoxide radical (O_2_^•−^) and singlet oxygen (^1^O_2_) [5,6,7]. Various methods including UV, heating, ultrasound, carbonaceous materials and transition metals have been utilized to activate PMS and H_2_O_2_ [8,9,10]. Considering the economic benefits of no additional energy consumption and high removal efficiency (Table 1), transition metals modified plant-based biochar is an excellent candidate because of its wide source, transition metals, persistent free radicals and rich functional groups [11,12,13]. However, although it is effective, the preparation process of biochar consumes high energy and produces air pollutants, which will inevitably pollute the environment [14,15]. As the raw material of biochar, plant materials such as bamboo, and wheat stalks are composed of lignin, cellulose and hemicellulose, which are relatively stable in structure and rich in various surface functional groups [16,17,18]. Transition metals modified plant particles have the potential to activate H_2_O_2_ or PMS. This new environmentally friendly material can significantly reduce the material cost while retaining the high degradation performance of organic pollutants. However, there is no study on the degradation performance of modified plant materials for refractory organic pollutants.

Among transition metals, iron is a privileged activator because of its large environmental stock and relatively low toxicity [29,30]. Fe^0^ or Fe(Ⅱ) transfer electrons to H_2_O_2_ or PMS to generate free radicals [31,32]. However, the conversion rate of Fe(Ⅲ) to Fe(Ⅱ) is an important factor limiting the activation of H_2_O_2_ or PMS. Previous studies have shown that S^2−^ can effectively promote conversion [33,34]. Therefore, FeS-modified rape straw may efficiently activate H_2_O_2_ or PMS.

Compared to bulk particles or natural minerals, nanoscale FeS particles are unstable and can agglomerate rapidly in aqueous solutions [35], which may limit the removal efficiency and present challenges to their environmental applications. Recent studies also indicated that the introduction of stabilizing agents such as chitosan [36], hydroxymethyl cellulose [37], and tetrasodium of N,N-bis(carboxymethyl) glutamic acid [38] can effectively prevent the aggregation of particles and enhance their physical stability and removal efficiency. The bisphenol A degradation efficiency in the combination of nano-sized BiFeO_3_ with chelating agents had the order: EDTA > nitrilotriacetic acid > glycine > formic acid > tartaric acid > none chelating agent [39]. Furthermore, a chelating agent could significantly enhance the efficiency of Fenton-like reactions at neutral and alkaline conditions.

The primary concerns of this study were to verify the following questions: (i) can heterogeneous Fenton-like reaction with FeS be reinforced by EDTA over various pH conditions; (ii) can the rape straw be efficient for the support and recycling of FeS and (iii) what the underlying mechanism of the activation process is. Based on the above consideration, this research was originally conducted to: (1) investigate the effects of various operating parameters including H_2_O_2_ dosage, PMS dose, pH values, and OTC concentration on OTC degradation, (2) evaluate the application potential of the catalysts by reusability and universal applicability, (3) shed light on the main reactive oxygen species and explain the crucial role of Fe(Ⅱ) in the oxidative process. This work is dedicated to utilizing rape straw waste in a valuable manner and simultaneously achieving the elimination of pollutants.

## 2. Materials and Methods

### 2.1. Samples and Reagents

Rape straw is obtained from a farm located in Chengdu, China. It is air-dried and meshed through 100 meshes. Iron sulfate heptahydrate (FeSO_4_·7H_2_O), sodium sulfide (Na_2_S·9H_2_O), 30% hydrogen peroxide (H_2_O_2_), potassium monopersulfate triple salt (KHSO_5_·0.5KHSO_4_·0.5K_2_SO_4_), 2,2,6,6–tetramethylpiperidine (TEMP), 5,5–dimethyl–1–pyrroline–N–oxide (DMPO), and ethylenediaminetetraacetic acid disodium salt (EDTA) were of reagent grade (Sichuan Kelong Industrial Group, Chengdu, China). OTC was purchased from Solarbio (Beijing, China).

### 2.2. Preparation of the Catalysts

FeS nanoparticles modified rape straw composites encapsulated with ethylenediaminetetraacetic acid (RS–EDTA–FeS) were prepared following a modified method. Distilled water was firstly purged with purified N_2_ (>99%) for 1 h to remove dissolved oxygen, and FeSO_4_·7H_2_O (12.64 g) was dissolved with strong magnetic stirring and N_2_ purging, EDTA (4.00 g) was added to the solution to form Fe^2+^–EDTA complexes. Then, rape straw particles (4.00 g) were introduced into the mixture, and FeS particles were synthesized on the surface of the rape straw by dropwise addition of Na_2_S·9H_2_O solution (10.91 g), then the suspension was sealed and aged for 12 h. FeS nanoparticles modified rape straw composites (RS–FeS) were prepared in the same as RS–EDTA–FeS without EDTA. The precipitations were freeze-dried for subsequent uses (Figure S1).

### 2.3. The Characterization of Catalysts

The specific surface area, total pore volume, and average pore size of RS–FeS and RS–EDTA–FeS was determined by N_2_ adsorption–desorption (ASAP2020, Micromeritics, Norcross, GA, USA) at 77 K. The Brunauer–Emmett–Teller (BET) model was employed to measure the specific surface areas of RS–FeS and RS–EDTA–FeS. Scanning electron microscope and energy dispersive spectrometer (SEM–EDS, JSM–7610F) was used to analyze the morphology and elemental composition. The types of chemical bonds and functional groups on the surface of RS–FeS and RS–EDTA–FeS were recorded on a Fourier transform infrared spectrometer (FTIR, Nicolet Avatar 660, Thermo, Nicolet, Madison, Wisconsin, WI, USA). The samples were mixed with KBr in a mass ratio of 1:100, and then followed were tested at a resolution of 2 cm^−1^ within a range of 4000–400 cm^−1^. X-ray diffraction (XRD, Ultima IV X-ray diffractometer, Rigaku, Japan) was performed using Cu Kα radiation at a voltage of 30 kV and current of 10 mA over in the 2*θ* range of 5–80° at a rate of 4°/min to analyze the crystal on the surface of catalysts. JADE V6.1 was used for data interpretation. Electron paramagnetic resonance spectroscopy (EPR, E500, Bruker, Germany) was adopted to determine the free radicals produced from the activation process using two spin-trapping reagents of DMPO and TEMP (100 mM). X-ray photoelectron spectroscopy (XPS, Quantum 2000 ESCA spectrometer) was recorded from an energy range of 0–1400 eV to analyze the elemental composition of the catalyst surfaces and valence states. The binding energy (BE) was calibrated by the C–C peak as a reference at 284.4 eV. The C 1s, O 1s, Fe 2p, and S 2p XPS spectra were determined by XPSPEAK 41 peak fitting software with a Gaussian/Lorentaian ratio of 80/20 after subtraction of a Shirley baseline.

### 2.4. Batch Experiments

#### 2.4.1. OTC Removal Comparison Experiments in Different Systems

To demonstrate the excellent catalytic performance of RS–FeS and RS–EDTA–FeS, the OTC removal in different reaction systems was compared in different systems. The adsorption capacity of RS–FeS and RS–EDTA–FeS were measured with 30.00 mL 20.00 mg L^−1^ OTC concentration at pH = 3.00 and 6.00. Furthermore, RS, FeS, EDTA–FeS, RS–FeS, and RS–EDTA–FeS were used to degrade OTC in PMS and H_2_O_2_ systems. These comparison experiments in H_2_O_2_ and PMS systems were conducted under 20.00 mg L^−1^ OTC at pH = 3.00 and 6.00.

#### 2.4.2. Single Factor Experiments

H_2_O_2_ and PMS activation experiments were conducted under different OTC concentrations (20.00, 40.00, 60.00, 80.00, and 100.00 mg L^−1^), solution pH (2.00, 4.00, 6.00, and 8.00), H_2_O_2_ dosage (6.60, 13.20, 19.80, and 26.70 mM), PMS concentration (0.10, 0.20, 0.40, 0.60, and 0.80 g L^−1^), and contact time (1.00, 2.00, 4.00, 10.00, 30.00, 60.00, and 120.00 min) to investigate the effects of reaction condition. CH_3_OH, Na_2_CO_3_, fulvic acid, and NaCl were applied to determine the effect of coexisting substances on OTC degradation.

OTC was dissolved in distilled water. The initial pH of the mixture was adjusted with 5% NaOH or HCl; 0.01 g RS–FeS or RS–EDTA–FeS were added to 30.00 mL OTC solution in 100 mL sealed glass bottles. After the reaction, catalysts were filtrated through 0.45–μm microporous membranes. OTC concentration was determined by a UV–vis Spectrophotometer at 354 nm.

#### 2.4.3. Cycling Experiments

The recycling test was conducted under optimal conditions. After the reaction, catalysts were separated and collected for the next degradation test under the same experimental conditions. After contact, the concentration of Fe^2+^ and total Fe in the solution was determined by the method of o–phenanthroline spectrophotometry.

#### 2.4.4. Treatment of Actual Wastewater

Actual wastewater was collected from an intensive pig farm (Chengdu, China). The wastewater was filtered and treated by the anaerobic–aerobic process. TOC was the index to testify to the removal of organic pollutants in wastewater. The experiments were conducted under the optimum condition of single-factor experiments.

## 3. Results and Discussion

### 3.1. Characterization of RS–FeS and RS–EDTA–FeS

**Figure 1:**
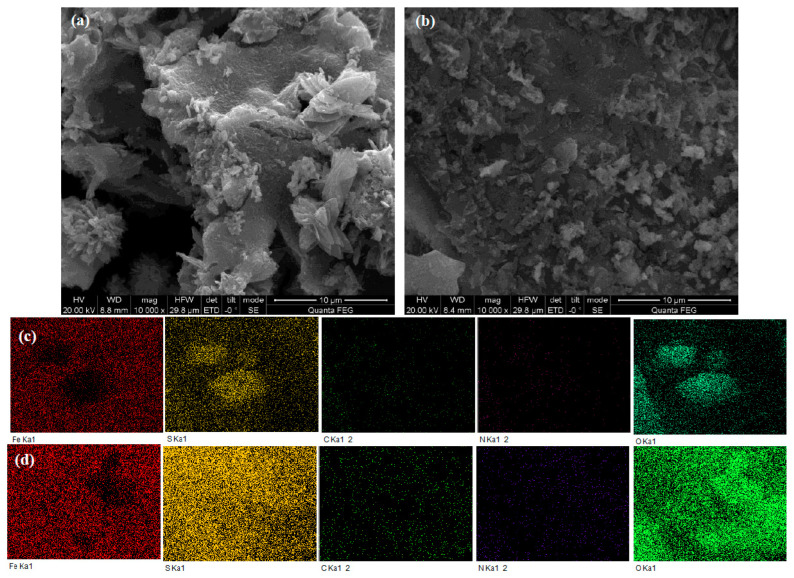
SEM images of RS–FeS (**a**) and RS–EDTA–FeS (**b**). EDS mappings of Fe, S, C, N, and O on RS–FeS (**c**) and RS–EDTA–FeS (**d**).

The microstructures and morphologies of catalysts were revealed by SEM–EDS. As illustrated in Figure 1, the images of pristine catalysts indicated that granular FeS nanoparticles were loaded on the surface of the rape straw. There are large rhombic crystals on the surface of RS–FeS and small irregular crystals on the surface of RS–EDTA–FeS, which indicates that the addition of EDTA effectively controls the crystal size. Furthermore, Fe and S have the same distribution characteristics in mapping results, indicating that FeS may be successfully synthesized on the surface of catalysts.

**Figure 2:**
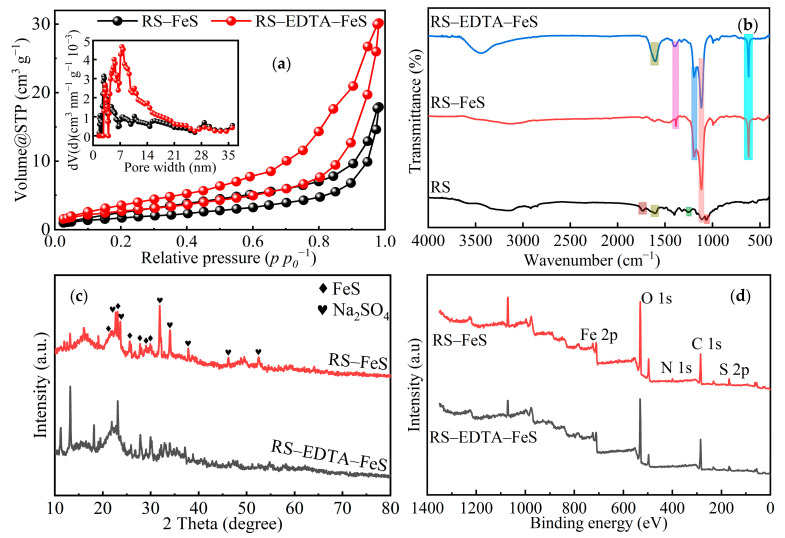
Nitrogen adsorption–desorption isotherms (**a**), FTIR spectra (**b**), XRD pattern (**c**), and XPS wide scan spectra analysis (**d**) of RS–FeS and RS–EDTA–FeS.

According to the BET method (Figure 2a), the specific surface areas of RS–FeS and RS–EDTA–FeS were 6.40 and 10.01 m^2^ g^−1^, which revealed that EDTA increased the specific surface of the catalyst. Li and Zhang (2021) reported that the surface area of pristine rape straw was 2.50 m^2^ g^−1^ [40]. Compare with pristine rape straw particles, the load of FeS significantly increased the specific surface [41]. Two catalysts demonstrated type Ⅲ isotherms, showing representative mesoporous structures [19]. These results were in line with pore size distribution. The pore size distribution curve revealed that the pore volume RS–FeS and RS–EDTA–FeS are mainly at about 0.023 and 0.042 cc g^−1^, the pore width of RS–FeS and RS–EDTA–FeS mainly distribute at 2.769 and 7.452 nm based on the DFT method summary.

The functional groups of catalysts were analyzed by FTIR (Figure 2b). RS–FeS and RS–EDTA–FeS have the same peaks at 617, 1119, and 1194 cm^−1^, which correspond to FeS and SO_4_^2−^ [41,42]. The results show that FeS is successfully loaded on the material surface. In addition, there may be sodium sulfate crystallization on the material surface. The symmetric and asymmetric stretching of –COOH corresponding to the peak of RS–EDTA–FeS at 1401 and 1603 cm^−1^ may come from EDTA [36,43]. There are abundant functional groups on the surface of rape straw, and the peaks at 1065, 1257, and 1738 cm^−1^ correspond to –OH, C–H and C=O groups, respectively [19,44]. After loading FeS, the peaks of –OH, C–H and C=O basically disappear, which may be because the functional groups are consumed by Fe in the ways of complexation, chelation, and redox.

The XRD patterns of RS–FeS and RS–EDTA–FeS are compared in Figure 2c. It can be seen that the peak of FeS was disordered and blunt, proving that the synthetic FeS had a poor crystallinity, existing in an amorphous and glassy state [41]. Obvious peaks at 2*θ* = 21.90°, 23.06°, 25.65°, 27.76°, 28.97°and 29.90° were indicative peak of FeS. Some obvious diffraction peaks of Na_2_SO_4_ appeared at 2*θ* = 22.67°, 23.67°, 31.75°, 34.15°, 37.80°, 46.21°, and 52.60°, which could be used as evidence for the formation of needle-shaped crystals in SEM [43].

**Figure 3:**
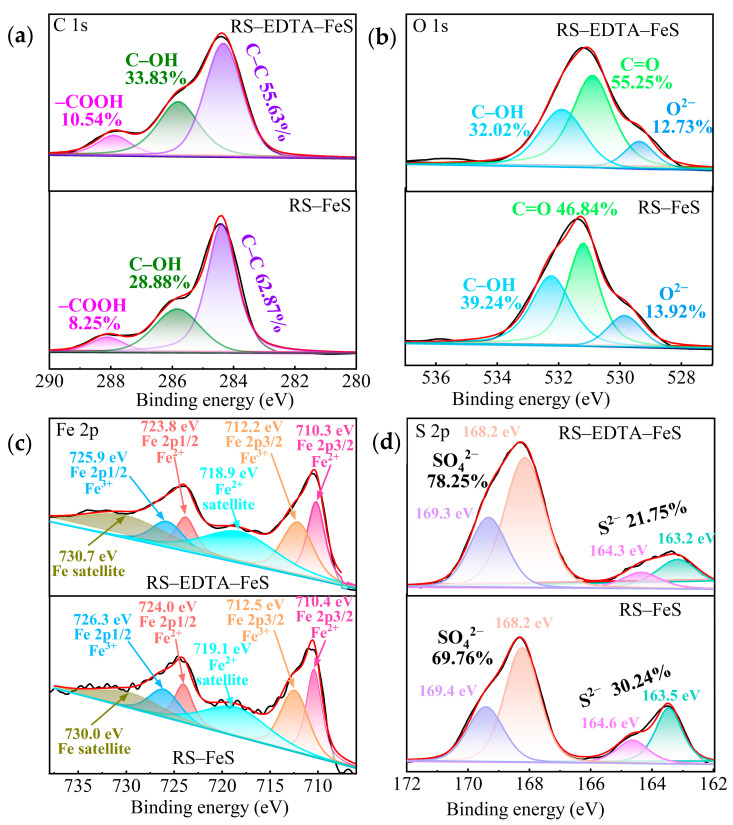
C 1s XPS spectra (**a**), O 1s XPS spectra (**b**), Fe 2p XPS spectra (**c**), and S 2p XPS spectra (**d**) of RS–FeS and RS–EDTA–FeS.

The XPS spectra of RS–FeS and RS–EDTA–FeS are shown in Figure 2d and Figure 3, which clearly demonstrate the oxygen-containing functional groups and the valence of Fe and S. The photoelectron peak of C 1s is divided into three peaks (Figure 3a). The binding energies approximately corresponded to 284.4, 285.8, and 287.9 eV representing C–C, C–O and O–C=O, respectively [45]. The O 1s spectrum was best fitted with three components at 529.4, 530.9, and 531.9 eV (Figure 3b), which correspond to O^2−^, C=O, and C–OH [19]. The results showed that the surfaces of the two catalysts were rich in oxygen-containing functional groups. Compared with RS–FeS, the addition of EDTA significantly increased the content of –COOH and –C=O.

The Fe 2p peaks observed at 710.1 and 712.1 eV, and 723.7 and 726.0 eV (Figure 3c) can be attributed to the Fe(Ⅱ) and Fe(Ⅲ) species in Fe 2p3/2 and Fe 2p1/2 [46,47]. The Fe(Ⅱ) contents of RS–FeS and RS–EDTA–FeS were 47.98 and 60.64%, indicating that the addition of EDTA significantly inhibited the oxidation of Fe(Ⅱ). The peaks observed at 163.2 and 164.4 eV, 168.2 and 169.3 eV (Figure 3d) correspond to S 2p3/2 and S 2p1/2 of S^2−^ and SO_4_^2−^ [41,48]. The S contents of RS–FeS and RS–EDTA–FeS were 30.24 and 21.75%, indicating that EDTA chelated with Fe(Ⅱ) may also inhibit the generation of FeS nanoparticles. These results revealed that both FeS and Na_2_SO_4_ nanoparticles loaded onto the surface of RS–FeS and RS–EDTA–FeS.

### 3.2. Degradation of OTC in Different Systems

To demonstrate the excellent catalytic performance of RS–FeS and RS–EDTA–FeS, the degradation of OTC in different reaction systems was compared. It is noted that the removal of OTC by RS–EDTA–FeS and RS–FeS alone at pH = 3.00 was 27.98 and 28.79% (Figure S2), suggesting that the adsorption of RS–EDTA–FeS and RS–FeS towards OTC were very weak.

The degradation rates of OTC in the RS/PMS and RS/H_2_O_2_ at 120 min were 22.88 and 25.46% (Figure S3), implying that the catalytic performance of RS was very poor. RS–FeS improved the degradation of OTC by PMS or H_2_O_2_, more than 80.00% of OTC was removed within 30.00 min. This proved that dispersing FeS onto the surface of RS is an efficient way to enhance the catalytic performance of RS [49]. In addition, OTC was also efficiently removed by FeS within 30 min, indicating that FeS exhibited facilitation in activating PMS and H_2_O_2_.

In this study, EDTA was introduced to inhibit the aggregation and oxidation of FeS nanoparticles. However, the OTC degradation efficiency of EDTA–FeS decreased by 30.00% as compared to FeS, implying EDTA significantly inhibited the activation performance of the catalyst, which can be attributed to the inhibition of electron transfer in the catalytic process by EDTA as an electron acceptor [39,50]. Furthermore, due to the steric hindrance, the rich chelating functional groups and the molecule size of EDTA restrained the Fe(II) accessibility. The accessible Fe(II) in the EDTA–Fe(II) complex was responsible for H_2_O_2_ and PMS activation and appropriate Fe(II) accessibility was favored for more efficient utilization of Fe(II) [51]. Among these catalysts, clearly, RS–EDTA–FeS also possessed excellent catalytic performance. Approximately 65.00% of OTC was degraded within 10.00 min. As compared with RS and FeS, RS–FeS and RS–EDTA–FeS with large surface area and pore volume provided more active sites, which was conducive to the activation of PMS and H_2_O_2_ for the OTC degradation.

### 3.3. Effects of Solution Chemistry on OTC Degradation by RS–FeS and RS–EDTA–FeS

#### 3.3.1. Solution pH

**Figure 4:**
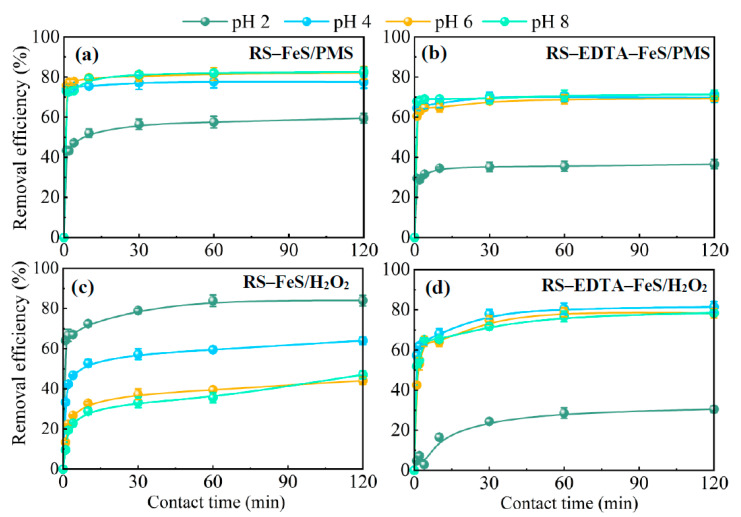
The effect of different initial pH on OTC removal efficiencies from the RS–FeS/PMS (**a**), RS–EDTA–FeS/PMS (**b**), RS–FeS/H_2_O_2_ (**c**), and RS–EDTA–FeS/H_2_O_2_ (**d**) system (C_0_ = 20.00 mg L^−1^, [PMS] = 0.20 g L^−1^, [H_2_O_2_] = 6.60 mM).

The catalytic reactivity of RS–FeS and RS–EDTA–FeS was investigated under various pH values in the PMS or H_2_O_2_ reaction system. As shown in Figure 4, OTC degradation efficiency first increased and then remained with the pH value increasing from 2.00 to 8.00 in the PMS system, indicating that RS–FeS and RS–EDTA–FeS can efficiently remove OTC in water at neutral pH, it is indispensable for the real application.

With the addition of H_2_O_2_, more than 80.00% of the OTC was degraded by RS–EDTA–FeS within 30.00 min in pH values ranging from 4.00 to 8.00. In the present work, the working pH of RS–EDTA–FeS can be extended to a neutral environment and retain excellent catalytic performance. RS–EDTA–FeS is indispensable for the real application of Fenton to allow a wider pH range of work. EDTA could form chelated complexes (Fe^2+^–EDTA) that could be used to maintain the iron soluble. Chelation is useful to extend the pH range over which iron is soluble because the chelating ligand competes favorably with hydroxide ion for coordination and chelated complexes are typically soluble [52].

Two catalysts activate PMS faster than H_2_O_2_, and the degradation rate of OTC reaches optimum in 4.00 min in the PMS system, while it took 60.00 min to get the equilibrium in the H_2_O_2_ system. However, the OTC degradation of the H_2_O_2_ system is higher than that of the PMS system. The maximum OTC degradation of RS–FeS and RS–EDTA–FeS observed in the H_2_O_2_ system were 87.51 and 81.15%. Two catalysts in this study overcome the shortcomings of the traditional Fenton reaction under acidic conditions, which will reduce the cost of adjusting the pH during the operation of the Fenton reaction.

#### 3.3.2. OTC Concentration

**Figure 5:**
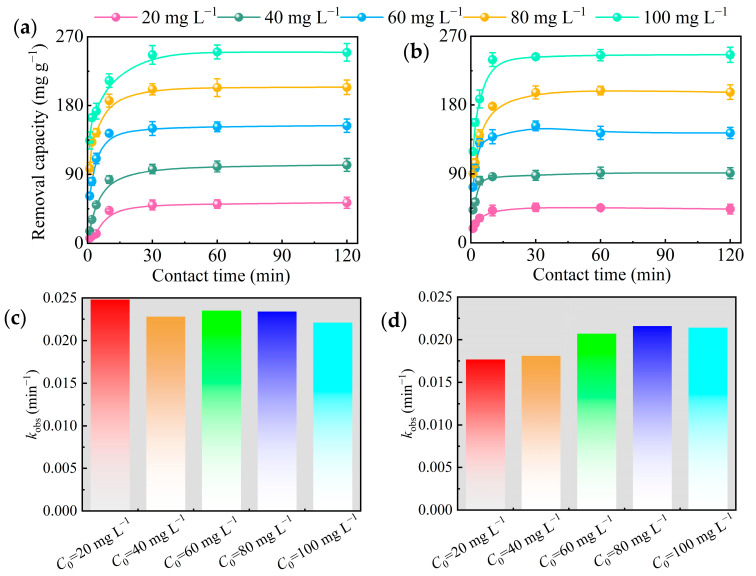
Effect of initial OTC concentration on OTC removal from the RS–FeS/H_2_O_2_ (**a**) and RS–EDTA–FeS/H_2_O_2_ (**b**) system. Kinetic data of OTC by RS–FeS/H_2_O_2_ (**c**) and RS–EDTA–FeS/H_2_O_2_ (**d**) system under different initial OTC concentration (initial pH = 3.00, [H_2_O_2_] = 6.60 mM).

Two catalysts were added to different concentrations of OTC solution to explore the maximum degradation. The degradation increased with the increase in OTC concentration, and the maximum amount in RS–FeS/H_2_O_2_ RS–EDTA–FeS/H_2_O_2_ RS–FeS/PMS and RS–EDTA–FeS/PMS system observed at 100.00 mg L^−1^ OTC was 251.07, 244.87, 218.84, and 214.52 mg g^−1^ (Figure S4 and Figure 5), which indicated that the two catalysts had the potential for long–term and sustained degradation of organic matter. The degradation performance toward OTC of RS–FeS is superior to RS–EDTA–FeS. RS–FeS exposed more active sites than RS–EDTA–FeS and reacts faster with PMS or H_2_O_2_. Moreover, Fe(II) is readily oxidized in an oxygen-rich condition, which leads to high storage costs. In addition, EDTA reduced the catalytic activity of RS–EDTA–FeS but effectively inhibited the oxidation of Fe(Ⅱ). As shown in Figure S1, RS–FeS changed from black to brown in 30 days, indicating that FeS was oxidized to Fe_2_O_3_, but RS EDTA FES remained black.

#### 3.3.3. PMS Concentration

**Figure 6:**
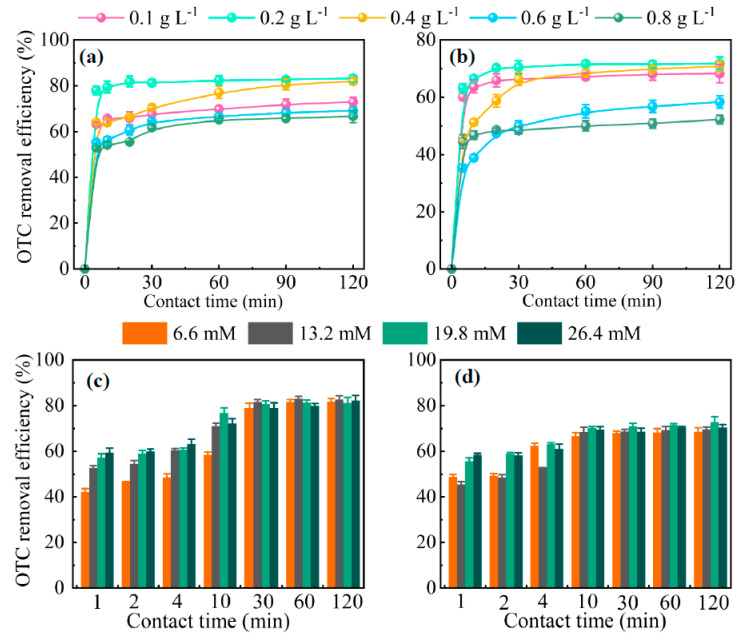
The effect of PMS concentration (**a**,**b**) and H_2_O_2_ dosage (**c**,**d**) on OTC removal by RS–FeS (**a**,**c**) and RS–EDTA–FeS (**b**,**d**) (C_0_ = 20.00 mg L^–1^, initial pH in H_2_O_2_ system = 3.00, initial pH in PMS system = 6.00).

As shown in Figure 6a,b, the addition of PMS also significantly affected the degradation of OTC. The degradation of OTC by RS–FeS and RS–EDTA–FeS firstly increased (0.10–0.20 g L^−1^) and then decreased (0.20–0.80 g L^−1^) with the addition of PMS. The optimal concentration of PMS was 0.20 g L^−1^. With the increase in PMS concentration from 0.20 mg L^−1^ to 0.80 mg L^−1^, the removal rates of OTC by RS–FeS and RS–EDTA–FeS decreased from 83.57 and 71.75% to some 66.65 and 52.12%. The reason for this was that the excess PMS scavenged free radicals [34].

#### 3.3.4. H_2_O_2_ Dosage

Various H_2_O_2_ dosages were tested to determine the optimal experimental conditions in Figure 6c,d. Accompanied by the enhancement of H_2_O_2_ dosage, the degradation rate of OTC by two catalysts first increased (6.60–19.80 mM) and then decreased (26.40 mM), the optimal H_2_O_2_ dosage for the degradation of OTC was 19.80 mM. More ^•^OH was produced with the increase in H_2_O_2_, but excessive H_2_O_2_ may inhibit the transfer of free radicals [53].

#### 3.3.5. Coexisting Substances

**Figure 7:**
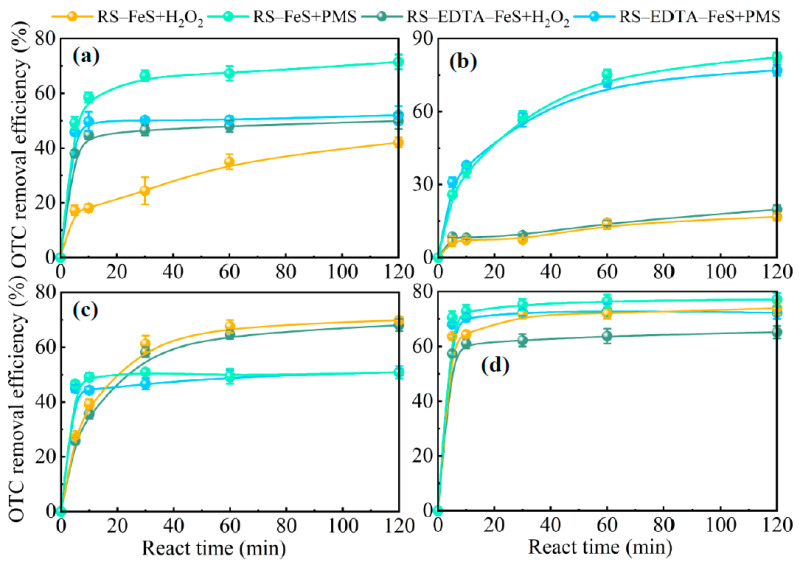
The effect of coexisting CH_3_OH (**a**), Na_2_CO_3_ (**b**), fulvic acid (**c**), and NaCl (**d**) substances on OTC degradation from the RS–FeS/PMS, RS–EDTA–FeS/PMS, RS–FeS/H_2_O_2_, and RS–EDTA–FeS/H_2_O_2_ system (C_0_ = 20.00 mg L^−1^, [PMS] = 0.20 g L^−1^, [H_2_O_2_] = 6.60 mM, initial pH in H_2_O_2_ system = 3.00, initial pH in PMS system = 6.00).

In this study, the effects of CH_3_OH, Na_2_CO_3_, NaCl, and fulvic acid on the degradation of OTC in the RS–FeS/PMS, RS–EDTA–FeS/PMS, RS–FeS/H_2_O_2_, and RS–EDTA–FeS/H_2_O_2_ system were investigated, and the results are shown in Figure 7. CH_3_OH significantly inhibited the degradation of OTC in the H_2_O_2_ system. Previous studies have shown that CH_3_OH can quench ^•^OH [54]. This result shows that ^•^OH may be the key ROS to degrade OTC in the H_2_O_2_ system. As shown in Figure 7b, the addition of Na_2_CO_3_ retarded the degradation of OTC obviously at the initial stage of the reaction in the PMS system, which could be attributed to the fact that the introduction of Na_2_CO_3_ quenched O_2_^−^. In addition, Na_2_CO_3_ significantly restrained the degradation of OTC in the H_2_O_2_ system by raising the solution pH from 3.00 to 5.00. Raising pH weakened the electrostatic attraction between the catalyst and H_2_O_2_ or OTC. Fulvic acid is a natural organic matter, and its effect on the degradation of OTC is also shown in Figure 7c. It has been reported that fulvic acid could compete with OTC for SO_4_^•−^ and ^•^OH, resulting in a low degradation efficiency of OTC. However, the degradation efficiency of OTC in our research just has a slight decrease, further confirming that SO_4_^•−^ and ^•^OH were not the main active species. Furthermore, NaCl slightly repressed the OTC removal in both H_2_O_2_ systems in Figure 7d. It has been reported that Cl^−^ can react with SO_4_^•−^ and ^•^OH to generate Cl· with lower oxidation ability [55], which would weaken the degradation of OTC. However, the introduction of Cl^−^ did not exert a significant effect on the degradation of OTC in the PMS system. Thus, it is speculated that SO_4_^•−^ and ^•^OH may not be the main ROS responsible for the OTC degradation in the reaction system (further discussed later).

### 3.4. Identify the Active Species and Reaction Mechanism

**Figure 8:**
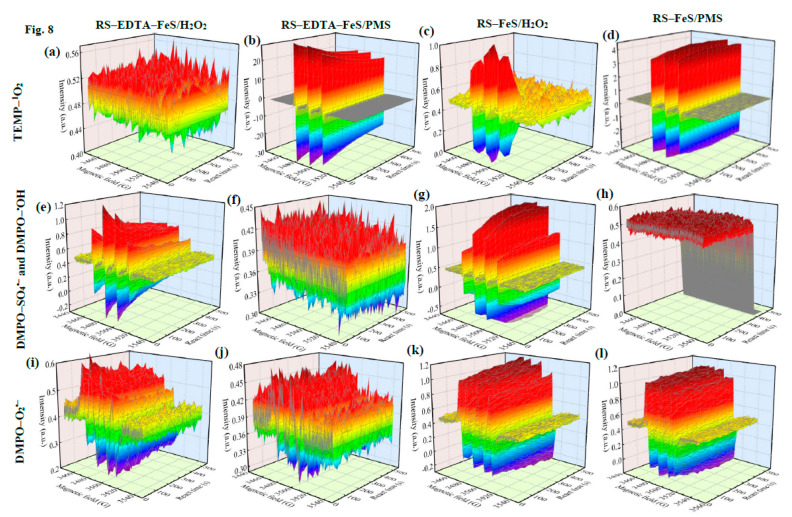
EPR spectrum on radicals in RS–FeS/PMS, RS–EDTA–FeS/PMS, RS–FeS/H_2_O_2_, and RS– EDTA–FeS/H_2_O_2_ systems captured by TEMP and DMPO. ^1^O_2_ in RS–EDTA–FeS/H_2_O_2_ (**a**), RS–EDTA–FeS/PMS (**b**), RS–FeS/H_2_O_2_ (**c**), and RS–FeS/PMS (**d**) systems; SO_4_^•–^ and ^•^OH in RS–EDTA–FeS/H_2_O_2_ (**e**), RS–EDTA–FeS/PMS (f), RS–FeS/H_2_O_2_ (**g**), and RS–FeS/PMS (**h**) systems; O_2_^•–^ in RS–EDTA–FeS/H_2_O_2_ (**i**), RS–EDTA–FeS/PMS (**j**), RS–FeS/H_2_O_2_ (**k**), and RS–FeS/PMS (**l**) systems.

In order to explore the difference between the two catalysts in H_2_O_2_ or PMS system, ESR spectra were tested at different time points to build a 3D map. It revealed the change of ROS at different times in the reaction process. As shown in Figure 8, TEMP–^1^O_2_ signals (three–line peaks, intensities of 1:1:1), DMPO–O_2_^•–^ signals (four-line peaks, intensities of 1:1:1:1), DMPO–SO_4_^•–^ signals (six–line peaks, intensities of 1:1:1:1:1:1), and DMPO–^•^OH signals (four-line peaks, intensities of 1:2:2:1) were detected in the ESR spectra.

The major reactive oxygen species of the two catalysts in the PMS or H_2_O_2_ system were different. O_2_^•–^ and ^1^O_2_ were major ROS in the PMS system. It has been reported that carbonaceous materials easily activate PMS to produce ^1^O_2_ [56]. ^1^O_2_ is mainly derived from carbonyl carbon (C=O) interacting with PMS. Furthermore, Fe_2_O_3_ on the surface of the catalyst can cause lattice distortion, promoting the release of active oxygen and accelerating ^1^O_2_ generation [57]. Previous studies have shown that singlet oxygen is the main active substance of the PMS system [3,8]. Furthermore, the generation of ^1^O_2_ has been stable with time, which provides the possibility of continuous and efficient degradation of organic pollutants. O_2_^•–^ can also be generated during PMS self–decomposition under acidic or neutral conditions. However, O_2_^•–^ can be easily recombined to ^1^O_2_.

Instead, O_2_^•–^ and ^•^OH were the major ROS in the H_2_O_2_ system. The appearance of the DMPO–O_2_^•–^ signal could be ascribed to the fast transformation of ^•^OH to·O_2_^•–^ on the high–activity and metastable active sites of the RS–FeS and RS–EDTA–FeS. As H_2_O_2_ concentrations increase, ·OH reacts with the excess H_2_O_2_ to generate O_2_^•–^. Furthermore, the formed O_2_^•−^ could react with H_2_O and ^•^OH to produce ^1^O_2_. Figure 8 demonstrated that the typical triplet peak of TEMP–^1^O_2_ (1:1:1) appeared and became stronger over time, revealing an increasing amount of ^1^O_2_ was produced during the H_2_O_2_ activation. These results identified the difference between PMS and H_2_O_2_ reaction systems, and further demonstrated the effect of EDTA.

### 3.5. Reusability of RS–FeS and RS–EDTA–FeS

**Figure 9:**
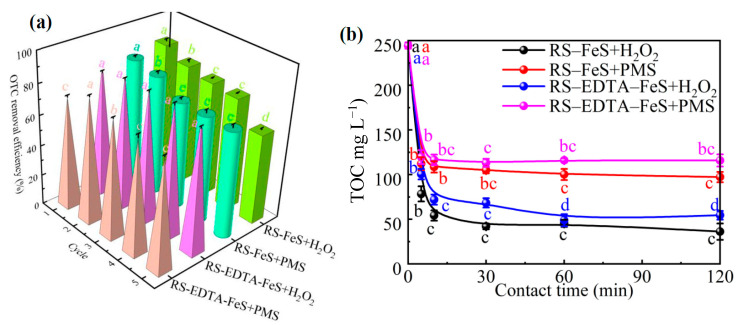
(**a**) Cycling test of RS–FeS and RS–EDTA–FeS for OTC removal in PMS and H_2_O_2_ systems (C_0_ = 20.00 mg L^−1^, [PMS] = 0.20 g L^−1^, [H_2_O_2_] = 6.60 mM, initial pH in H_2_O_2_ system = 3.00, initial pH in PMS system = 6.00, [t] = 120.00 min). (**b**) TOC removal by RS–FeS and RS–EDTA–FeS in PMS and H_2_O_2_ systems from swine wastewater. (Initial TOC concentration = 244.3 mg L^−1^, [PMS] = 0.20 g L^−1^, [H_2_O_2_] = 6.60 mM, initial pH in H_2_O_2_ system = 3.00, initial pH in PMS system = 6.00). Error bars represent the standard deviations (*n*=3); different lowercase letters represent significant difference between different treatments (*P* < 0.05).

The stability of two catalysts was evaluated in successive experiments to reveal the stability and environmental risk of Fe(Ⅱ/Ⅲ) (Figure 9a). RS–FeS and RS–EDTA–FeS were recycled through five experiments and their catalytic activities were measured. The OTC degradation efficiency of RS–FeS gradually decreased under recycling. At the fifth recycle, the removal efficiency of RS–FeS in PMS and H_2_O_2_ systems decreased from 82.00 and 86.00% to 70.00 and 59.00%, respectively. The decrease may be attributable to the loss of Fe and S during the cyclic reaction process. On the contrary, RS–EDTA–FeS maintained excellent efficiency and stability in each subsequent experiment. The degradation efficiency of OTC did not decrease significantly after five cycles of RS–EDTA–FeS in the H_2_O_2_ and PMS systems. The degradation efficiency of OTC of RS–EDTA–FeS in PMS system even increased under reuse. Above results identified that the addition of EDTA increases the reuse feasibility of RS–EDTA–FeS.

The difference between total iron and Fe(Ⅱ) in the solution after the reaction was tested to demonstrate the environmental risk of the two catalysts. As illustrated in Figure S5, the concentration of total iron gradually decreases under recycling, and Fe(Ⅲ) was dominant in the solution. In addition, the Fe(Ⅱ) first decreased and then increased in the H_2_O_2_ system with the increase in reaction times. Combined with the OTC degradation performance of the two catalysts in the H_2_O_2_ system, Fe(Ⅱ) may not be the main effect on the Fenton reaction. In a further study, reducing the load of FeS may be an effective way to reduce environmental risk and maintain degradation efficiency.

### 3.6. Practical Application and Cost Estimation

In order to evaluate the practical value of the two catalysts, TOC removal of preliminarily treated swine wastewater was compared in PMS or H_2_O_2_ system. As shown in the Figure 9b, the TOC removal of RS–FeS and RS–EDTA–FeS in the H_2_O_2_ system was 85.23 and 77.70%, the TOC removal of RS–FeS and RS–EDTA–FeS in the PMS system was 60.24 and 52.58%, indicating that RS–FeS was the best choice to activate H_2_O_2_ and remove TOC from actual wastewater. The result is also consistent with the previous research.

The abovementioned research clearly demonstrated the feasibility of RS–FeS and RS–EDTA–FeS for oxytetracycline degradation. The cost of treatment was further determined for the processes applied for the degradation of oxytetracycline. The cost estimation of RS–FeS/H_2_O_2_, RS–FeS/PMS, RS–EDTA–FeS/H_2_O_2_, and RS–EDTA–FeS/PMS system for oxytetracycline degradation consisted of three parts: (i) the cost of RS preparation; (ii) the cost of RS–FeS and RS–EDTA–FeS catalyst and (iii) cost of the oxidants. From a previous experimental study, we estimated the total cost of these four systems of treating each liter of wastewater was about 0.60 CNY (Table S1). Hence, the process presented a promising industrial application potential.

## 4. Conclusions

In this work, a comprehensive study was conducted to investigate the degradation behavior of OTC by FeS and EDTA-modified rape straw in the Fenton–like process. The results revealed that RS–FeS and RS–EDTA–FeS were excellent heterogeneous catalysts and possessed high activation ability of H_2_O_2_ and PMS for the efficient generation of ^1^O_2_, O_2_^•–^, ^•^OH or SO_4_^•–^ to degrade target pollutants. Compared with PMS, two catalysts in the H_2_O_2_ system have better performance on OTC degradation. Furthermore, the optimal pH of two catalysts in the H_2_O_2_ system was neutral; it is beneficial to decrease the corporation cost of the Fenton process. In addition, EDTA inhabited the activation of RS–EDTA–FeS, but the stability and reusability of RS–EDTA–FeS were prolonged by EDTA. Furthermore, EDTA restrained the oxidization of Fe(Ⅱ), extending the expiration date of RS–EDTA–FeS. Therefore, RS–EDTA–FeS in the H_2_O_2_ system was the best way to remove organic matter in our comparative study.

This study proves that the modified plant materials can activate H_2_O_2_ or PMS efficiently. Compared with biochar, plant materials like rape straw are low-cost and less polluting. This study broke the dominance of biochar in the research process of advanced oxidation and provided a new theoretical reference for the operation of a Fenton-like reaction with high efficiency and low cost.

## Figures and Tables

**Table 1:** Plant-based material activated different oxidant for the degradation of various emerging organic contaminants.

Pollutants	Catalysts	Oxidant	Optimal Experiment Terms	Degradation Capacity	Ref.
Tetracycline hydrochloride	Magnetic rape straw biochar (MRSB)	Persulfate	Persulfate content 8.00 mM; MRSB dosage 1.00 g/L; Reaction temperature 25℃; Initial pH 5.68	99.00%	[19]
Reactive black 5	Straw-iron composite material (ST@Fe)	Persulfate	Initial concentration 20.00 mM; Contact time 100.00 min; ST@Fe dosage 0.50 g/L	>94.80%	[20]
Acid Red 1	Iron-loaded rice husk biochar	H_2_O_2_	Initial concentration 50.00 mg/L; H_2_O_2_ concentration 16.00 mM; pH 3.00	98.00%	[21]
Tetracycline	Bagasse biochar modified with cobalt-iron	Peroxymonosulfate	Initial concentration 20.00 mg/L; Catalyst dose 0.30 g; Peroxymonosulfate concentration 0.40 g/L; Contact time 30.00 min; pH 5.00	96.70%	[22]
Sulfamethoxazole	Coconut shell biochar/C_3_N_4_ doped with magnetic oxygen	Peroxymonosulfate	Initial concentration 0.04 mM; Peroxymonosulfate concentration 1.60 mM; Catalyst does 0.40 g/L; pH 3.00	99.50%	[23]
Tetracycline	Cobalt and iron coloaded pomelo peel biochar	Peroxymonosulfate	Initial concentration 50.00 mg/L; Peroxymonosulfate concentration 1.00 g/L; Catalyst dose 0.10 g/L; pH 3.00	99.50%	[24]
Sunset Yellow	Fe-embedded waste coffee biochar	H_2_O_2_	Initial concentration 10.00 mg/L; H_2_O_2_ concentration 5.00 mM; Catalyst dose 0.40 g/L; pH 3.00	93.00%	[25]
Phenol	Citrus peels biochar	Peroxymonosulfate	Catalyst dose 0.20 g/L; Peroxymonosulfate dosage 3.20 mM; Reaction time 60.00 min	100%	[26]
Tetracycline	NaOH-modified Platanus orientalis Linn branches biochar	Peroxymonosulfate	Initial tetracycline concentration 0.02 g/L; Catalyst dose 0.50 g/L; Peroxymonosulfate dosage 0.50 mM; pH 4.79	97.90%	[27]
Oxytetracycline	Co_3_O_4_-corn straw hierarchical porous nanosheets	Peroxymonosulfate	Initial oxytetracycline concentration 40.00 μM; Peroxymonosulfate concentration 0.50 mM; Catalyst dose 0.20 g/L; pH 5.00; Reaction temperature 20 °C	100.00%	[28]

## Data Availability

The data presented in this study are available on request from the corresponding author.

## Supplementary Materials

The following supporting information can be downloaded at: https://www.mdpi.com/article/10.3390/molecules28062771/s1, Table S1. Cost estimation of RS–FeS and RS–EDTA–FeS for the degradation of oxytetracycline; Figure S1. RS-FeS and RS-EDTA-FeS after 30 days; Figure S2. OTC adsorption capacity of RS–FeS and RS–EDTA–FeS; Figure S3. Degradation of OTC in different systems. Experiments conditions: initial OTC concentration = 20.00 mg L^−1^; H_2_O_2_ = 20.00 սL; PMS = 0.20 g L^−1^; pH of PMS system = 6.00, pH of H_2_O_2_ system = 3.00; Figure S4. Effect of initial OTC concentration on OTC removal from the RS–FeS/PMS (a) and 115 RS–EDTA–FeS/PMS (b) system. Kinetic data of OTC by RS–FeS/PMS (c) and RS–EDTA– 116 FeS/PMS (d) system under different initial OTC concentration (initial pH=6.00, [PMS]=0.20 mg L^−1^); Figure S5. Total Fe and Fe(Ⅱ) leakage over 5 cycles; Table S1. Cost estimation of RS–FeS and RS–EDTA–FeS for the degradation of oxytetracycline.

## Sample Availability

Not available.
