# Supplementary material for: Rape Straw Supported FeS Nanoparticles with Encapsulated Structure as Peroxymonosulfate and Hydrogen Peroxide Activators for Enhanced Oxytetracycline Degradation
Source: Molecules. 2023 Mar 19;28(6):2771. doi: 10.3390/molecules28062771 (PMC10053016; doi:10.3390/molecules28062771)

Supplementary Material

Rape straw supported FeS nanoparticles with encapsulated structure as peroxymonosulfate and hydrogen peroxide activators for enhanced oxytetracycline degradation

Guiyin Wang [a, 1], Yan Yang [a, 1], Xiaoxun Xu [a, *], Shirong Zhang [a], Zhanbiao Yang [a], Zhang Cheng [a], Junren Xian [a], Ting Li [b], Yulin Pu [b], Wei Zhou [b], Gang Xiang [c], Zhien Pu [c]

[a] College of Environmental Sciences, Sichuan Agricultural University, Chengdu 611130, China.

[b] College of Resources, Sichuan Agricultural University, Chengdu 611130, China.

[c] College of Agronomy, Sichuan Agricultural University, Chengdu 611130, China

* Correspondence: Corresponding author at: College of Environmental Sciences, Sichuan Agricultural University, Wenjiang 611130, PR China. E–mail: xuxiaoxun@sicau.edu.cn; Fax: +86-028-8629-1233.

[1] These two authors contributed equally to this work.

Number of pages: 7

Number of Figures: 5

Number of Table: 1

Table S1 Cost estimation of RS–FeS and RS–EDTA–FeS for the degradation of oxytetracycline

Systems	The cost of RS preparation (electricity) (CNY/L)	The cost of catalyst preparation		The cost of the oxidants (CNY/L)	Total treatment cost (CNY/L)
		Reagent cost (CNY/L)	Cost of electricity (CNY/L)		
RS–FeS/H_2O_2	0.03	0.22	0.30	0.005	0.56
RS–FeS/PMS	0.03	0.22	0.30	0.03	0.58
RS–EDTA–FeS/H_2O_2	0.03	0.39	0.30	0.005	0.73
RS–EDTA–FeS/PMS	0.03	0.39	0.30	0.03	0.75

Fig. S1. RS-FeS and RS-EDTA-FeS after 30 days.

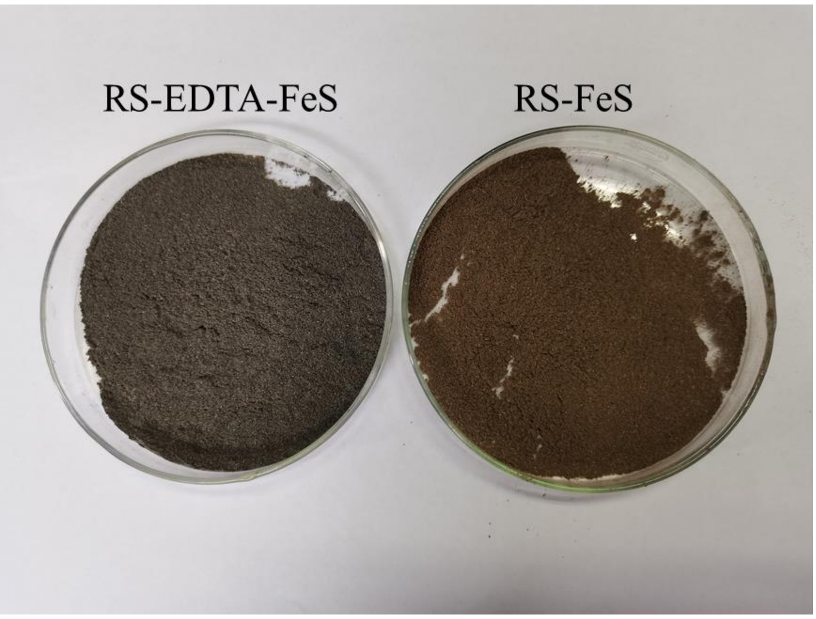

Fig. S2. OTC adsorption capacity of RS–FeS and RS–EDTA–FeS.

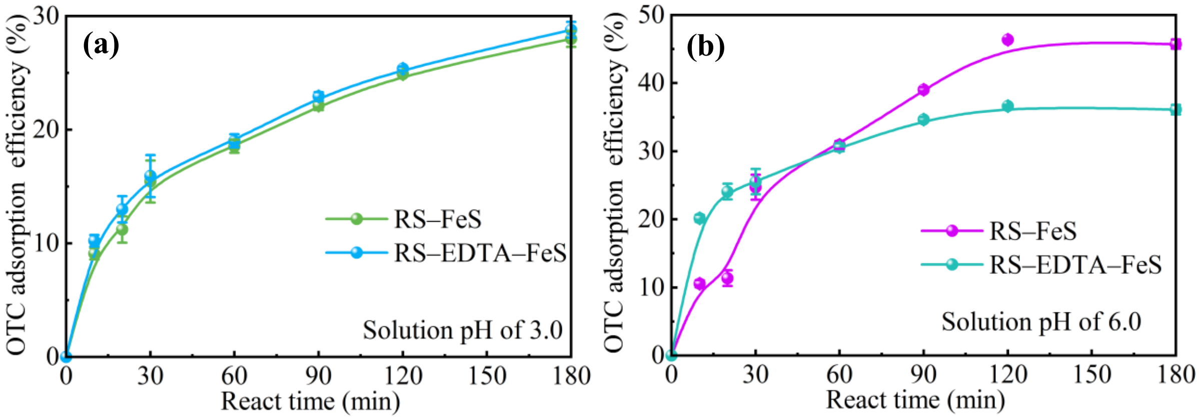

Fig. S3. Degradation of OTC in different systems. Experiments conditions: initial OTC concentration = 20.00 mg L^{-1}; H_2O_2 = 20.00 *u*L; PMS = 0.20 g L^{-1}; pH of PMS system = 6.00, pH of H_2O_2 system = 3.00.

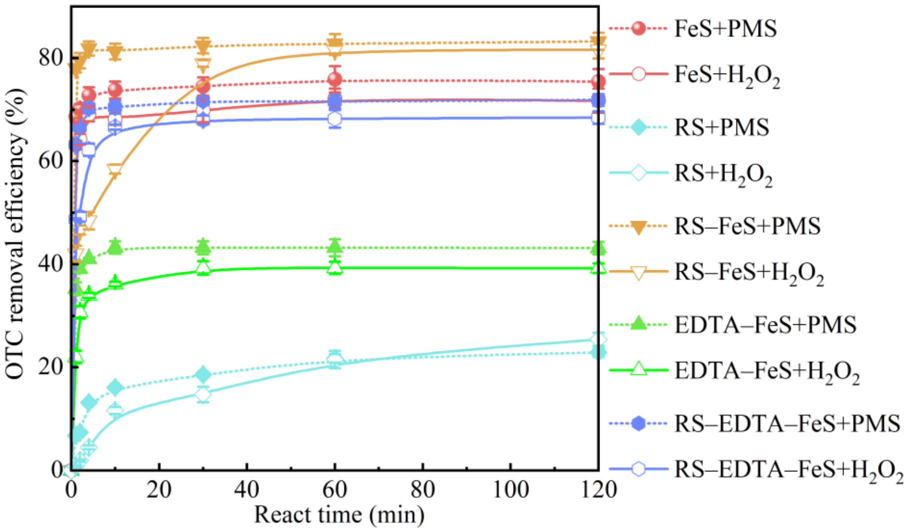

Fig. S4. Effect of initial OTC concentration on OTC removal from the RS–FeS/PMS (a) and RS–EDTA–FeS/PMS (b) system. Kinetic data of OTC by RS–FeS/PMS (c) and RS–EDTA–FeS/PMS (d) system under different initial OTC concentration (initial pH=6.00, [PMS]=0.20 mg L^{-1}).

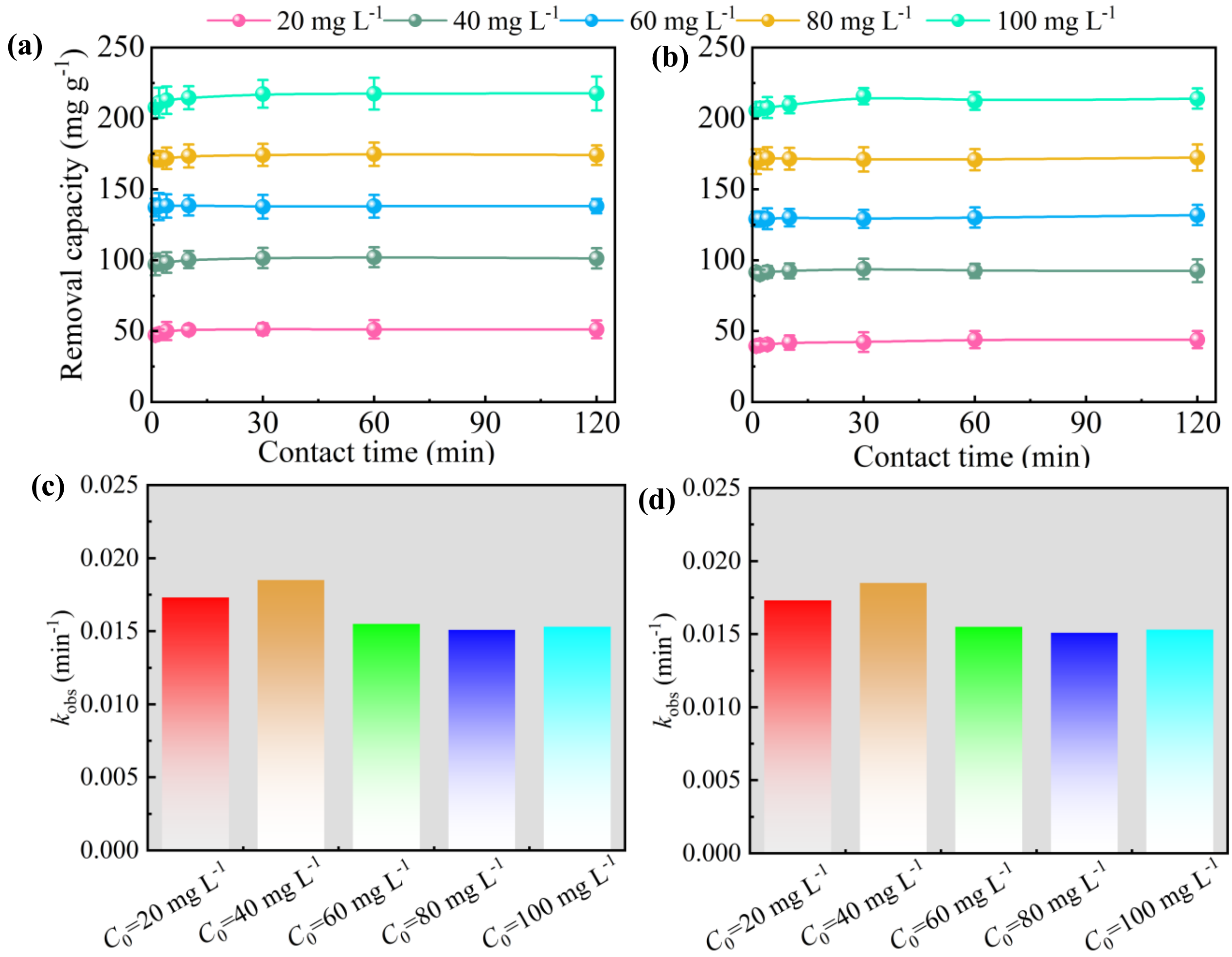

Fig. S5. Total Fe and Fe(Ⅱ) leakage over 5 cycles. Error bars represent the standard deviations (*n*=3); different lowercase letters represent significant difference between different treatments (*P* < 0.05).

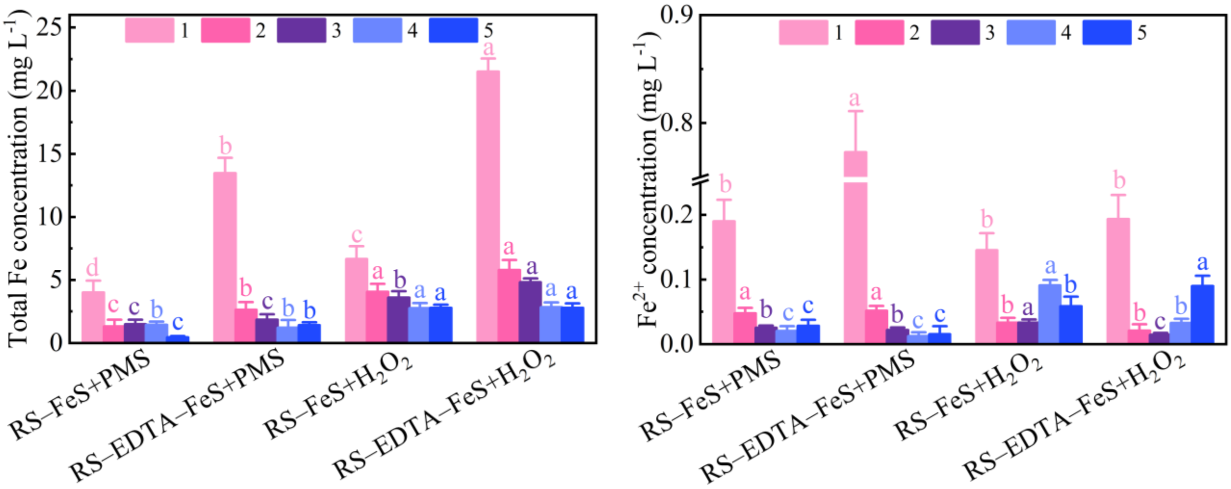

Supplement: Supplementary file 1 [file molecules-28-02771-s001.zip › molecules-2282634-supplementary.pdf]
